# Knock-down of LRP/LR promotes apoptosis in early and late stage colorectal carcinoma cells via caspase activation

**DOI:** 10.1186/s12885-018-4531-2

**Published:** 2018-05-29

**Authors:** Leila Vania, Thalia M. Rebelo, Eloise Ferreira, Stefan F. T. Weiss

**Affiliations:** School of Molecular and Cell Biology, University of the Witwatersrand, Private Bag 3, Wits 2050, Johannesburg, Republic of South Africa

**Keywords:** Colorectal cancer, Small interfering RNAs, Apoptosis, 37 kDa/67 kDa laminin receptor, LRP/LR, Therapeutics

## Abstract

**Background:**

Cancer remains one of the leading causes of death around the world, where incidence and mortality rates are at a constant increase. Tumourigenic cells are characteristically seen to over-express the 37 kDa/67 kDa laminin receptor (LRP/LR) compared to their normal cell counterparts. This receptor has numerous roles in tumourigenesis including metastasis, angiogenic enhancement, telomerase activation, cell viability and apoptotic evasion. This study aimed to expose the role of LRP/LR on the cellular viability of early (SW-480) and late (DLD-1) stage colorectal cancer cells.

**Methods:**

siRNA were used to down-regulate the expression of LRP/LR in SW-480 and DLD-1 cells which was assessed using western blotting. Subsequently, cell survival was evaluated using the MTT cell survival assay and confocal microscopy. Thereafter, Annexin V-FITC/PI staining and caspase activity assays were used to investigate the mechanism underlying the cell death observed upon LRP/LR knockdown. The data was analysed using Student’s t-test with a confidence interval of 95%, with *p*-values of less than 0.05 seen as significant.

**Results:**

Here we reveal that siRNA-mediated knock-down of LRP led to notable decreases in cell viability through increased levels of apoptosis, apparent by compromised membrane integrity and significantly high caspase-3 activity. Down-regulated LRP resulted in a significant increase in caspase-8 and -9 activity in both cell lines.

**Conclusions:**

These findings show that the receptor is critically implicated in apoptosis and that LRP/LR down-regulation induces apoptosis in early and late stage colorectal cancer cells through both apoptotic pathways. Thus, this study suggests that siRNA-mediated knock-down of LRP could be a possible therapeutic strategy for the treatment of early and late stage colorectal carcinoma.

**Electronic supplementary material:**

The online version of this article (10.1186/s12885-018-4531-2) contains supplementary material, which is available to authorized users.

## Background

Cancer remains one of the main causes of death around the world, where incidence and mortality rates are at a constant increase. According to the World Health Organisation (WHO), over 14 million new cases were diagnosed in 2015, and 8.8 million cancer related deaths were reported [1]. The current study focuses on a particular cancer type known as colorectal cancer. In South Africa, colorectal cancer is found to be the 5th most common cancer [2]. However, globally, it has been ranked as the 3rd most common cancer type with over 1.4 million new cases in the year 2015 – contributing to 9.7% of the total number of cancer cases diagnosed, including 774,000 cancer related deaths [1]. Due to the increasing prevalence and mortality rates of colorectal cancer, it is crucial to develop a novel treatment strategy to combat this disease.

There are several intrinsic and extrinsic factors which contribute to normal cells transforming into cancerous cells. Due to the complexity and diversity of neoplastic diseases, the collective term known as “the hallmarks of cancer” came about in order to provide a better understanding of this disease [3]. These hallmarks show that tumour cells acquire several capabilities that their normal counterparts do not have, including: independent of growth signals, resistance to anti-growth signals, unlimited replicative potential, tissue invasion and metastasis, continuous angiogenesis and apoptosis evasion [3]. In addition, recent studies have shown that cancerous cells also require the help of a particular receptor known as the 37 kDa laminin receptor precursor/67 kDa laminin receptor (LRP/LR) to maintain their tumourigenic state [4–9].

LRP/LR, also known as RPSA, is known to assist in numerous physiological processes [10, 11] . Moreover, the receptor possesses a strong binding affinity for laminin-1, a ligand found in several non-collagenous glycoproteins and is said to play critical roles in cell attachment, cell growth and differentiation [12], cell migration [13] and angiogenesis [14]. Hence, the interaction between LRP/LR and laminin-1 in is seen as an enhancement of tumour growth and progression [15]. In addition, LRP/LR has also been seen to play several other roles such as maintaining ribosomal processing of RNA [16], protein synthesis [17], cell cycle regulation [17] and importantly, cell survival [18].

Several studies have shown that LRP/LR contributes to many other pathological conditions such as microbial infections [19], neurological diseases including Alzheimer’s disease [20–22], prion-related diseases [23], as well as numerous other cancer types [10]. Furthermore, Naidoo et al. has also shown that LRP/LR mediates telomerase activity by enhancing hTERT activity, thus, illustrating a novel role for the receptor [24, 25]. Due to LRP/LR being involved in several of the aforementioned tumourigenic processes, this prompted the investigation of the receptor’s role in cellular viability and cell survival. One study has revealed that through silencing LRP/LR via siRNA technology, the viability of cervical (HeLa) [26], liver (Hep3B) [27] and lung (A549) [26] cancer cells was reduced by means of apoptotic induction. Other studies indicated that the viability of breast (MCF-7 and MDA-MB231) [28], oesophageal (WHC01) [28], neuroblastoma (IMR-32) [29], pancreatic (AsPC-1) [29] as well as malignant melanoma cancer cells [30] was also reduced through siRNA-mediated LRP/LR knockdown. Therefore, these studies show the vital role of LRP/LR in apoptosis and maintaining tumour cell survival.

Apoptosis is essential for several other processes within organisms including: tissue homeostasis maintenance, normal development preservation, as well as damaged cell elimination – all involving cells actively committing suicide. Once cells undergo apoptosis, they undergo several morphological and biochemical changes [31]. A biochemical change of importance to apoptosis is the activation of caspases. These caspases may become active through two key pathways and as a result induce apoptosis i.e. the intrinsic mitochondrial pathway and the extrinsic death receptor pathway [31].

Hence, the current study investigated whether siRNA-mediated knock-down of LRP/LR will reduce the viability of early (SW-480) and late (DLD-1) stage colorectal cancer cells. This study revealed that knock-down of LRP/LR using siRNA technology significantly reduces the viability of early and late stage colorectal cancer cells, and proposes that apoptosis is the cause for the notable decreases in cellular viability.

## Methods

A detailed list of suppliers/manufacturers of antibodies, reagents and equipment used to carry out the following experiments is given in the supplementary data section.

### Cell culture and conditions

Authenticated colorectal cancer cell lines SW-480 and DLD-1 were obtained from American Tissue Culture Collection (ATCC) with catalogue numbers ATCC® CCL-228 and ATCC® CCL-221, respectively. Both cancer cell lines were cultured in DMEM/Ham’s F-12 (1:1) (GE Lifesciences) together with 10% Fetal Calf Serum (FCS) (Capricorn Scientific) and 1% penicillin/streptomycin (Biowest). All cells remained at 37 °C with 5% CO_2_ in a humidified incubator.

### siRNA-mediated knock-down of the laminin receptor (LRP/LR)

Cell counts were performed with the TC20™ cell counter (Biorad) and cells were seeded at a density depending on the experiment being performed. Cells were allowed to reach 50–70% confluency prior to transfection. Both cancer cell lines were transfected with ON-TARGETplus SMARTpool Human-RPSA (GE Dharmacon) (targeted towards LRP/LR) – this siRNA will be referred to as siRPSA #1 and esiRNA-RLUC (serving as the negative control) (Sigma). The appropriate amounts of DharmaFect transfection reagent (GE Dharmacon) and Mission transfection reagent (Sigma) were added to the cells, respectively. The cancer cell lines were likewise transfected with esiRNA-RPSA (Sigma) which is also targeted to LRP/LR (this siRNA will be referred to as siRPSA #2). Thereafter, cells were incubated for 72 h at 37 °C. This procedure was performed before any further experiments took place.

### SDS-PAGE and western blotting

To determine siRNA-treated LRP/LR levels in the colorectal cancer cell lines, western blotting was performed. Cell lysates containing 10 μg of protein were separated on 12% sodium dodecyl sulphate polyacrylamide gels via electrophoresis (SDS-PAGE) (Bio-Rad). Thereafter, PVDF membranes (Pall Corporation) were soaked in methanol (Associate Chemical Enterprise, ACE) for 2 min followed by a 5-min incubation in transfer buffer. Proteins were transferred at 300 V via electro-blotting. The membranes were then blocked for an hour in 0.1% PBS-Tween in 3% BSA. Thereafter, the membranes were incubated with the IgG1-iS18 primary antibody which was diluted in blocking buffer (1:5000). The membranes were then washed three times in PBS-Tween (10 min for each wash) followed by an incubation in the appropriate secondary antibody diluted in blocking buffer (1:10000) for 1 h. The membrane was washed three more times with PBS-Tween before adding the chemiluminescent substrate (Biorad) to the membrane in order to detect proteins. In addition, 42 kDa β-actin served as a loading control. Finally, densitometric analysis was completed in order to quantify protein levels using ImageLab™ software.

### MTT assay

The MTT assay is a valid assay for determining cell viability employed in various cancer studies [28–30, 32–35]. Before transfections took place, 1 × 10^4^ cells/ml SW-480 and DLD-1 cells were seeded on 24-well plates. After transfection, cells were incubated at 37 °C for 72 h, followed by the addition of 1 mg/ml of MTT [100 μg of MTT (Duchfei Biochemic) being dissolved in 1 X PBS (Gibco)] to all wells. This was followed by a further incubation at 37 °C for 2 h. Thereafter, the media from each well containing MTT was removed and 500 μl DMSO was added to dissolve the residual formazan crystals (Merck Millipore). The resultant absorbance was measured at 570 nm. This procedure was performed for controls as well which included: untreated cells, PCA (Protocatechuic acid) positive control (Aldrich Chemistry) treated cells as well as esiRNA-RLUC negative control treated cells.

### Assessment of nuclear morphological changes – Confocal microscopy with Airyscan

In order to evaluate nuclear morphology post knock-down of LRP/LR via siRNA technology, confocal microscopy was used. Early and late stage colorectal cancer cells were seeded onto coverslips at a cell density of 1 × 10^5^ cells/ml. After transfection, the cells were fixed in 4% PFA (Associated Chemical Enterprise, ACE) for 15 min prior to 3 washes with PBS. Once remaining PBS was blotted off after washing, cells were then incubated with 0.1% Triton-X for 20 min for permeabilization of the cell membrane. The cells were then washed twice followed by the addition of DAPI nuclear stain (Sigma) diluted in PBS (1:100) onto each coverslip and incubated for 8 min in the dark. Once stained, the coverslips were washed twice in PBS prior to each coverslip being mounted on a microscope slide with fluoromount (Sigma). The microscope slides were left to set for 45 min in the dark, after which they were maintained at 4 °C. Note: untreated cells were used as a control, esiRNA-RLUC as a negative control and PCA as a positive control. Airyscan is a technique used to enhance confocal laser scanning microscopy. It has been shown that total resolution is improved by a factor of 1.7 in all spatial directions i.e. a resolution of 140 nm laterally and 400 nm axially can be achieved. The Airyscan was used to further analyse the nuclear morphological changes observed after LRP/LR down-regulation (Zeiss LSM 780).

### Annexin V-FITC/7AAD assays

This experiment was performed as per the manufacturer’s directions (Beckman Coulter). Both SW-480 and DLD-1 cells were seeded at a cell density of 2 × 10^6^ cells/ml before transfection. After 72 h incubation at 37 °C, cells were subjected to trypsinization with trypsin/EDTA (Biowest) followed by washes with cold PBS. Thereafter, cells were centrifuged at 5000 rpm for 5 min was performed, after which pellets were resuspended in 1X annexin-binding buffer (BD Sciences). Thereafter, 10 μl of Annexin V-FITC (BD Sciences) solution and 5 μl of PI viability dye were added to each cell suspension which was followed by a 15-min incubation on ice in the dark. Subsequently, 400 μl of ice-cold 1X annexin binding buffer was added to the samples for 30 min and all resulting cell suspensions were reviewed using the BD Accuri C6 flow cytometer. Note: esiRNA-RLUC was the negative control and PCA was the positive control.

### Caspase-3, − 8 and − 9 activation assays

Caspase-3,-8 and − 9 assays were completed as per the manufacturer’s directions (Merck Millipore). Cells were seeded at a cell density of 1 × 10^6^ cells/ml before transfection. Cells were then centrifuged at 1200 rpm for 10 min, followed by pellet resuspension in 50 μl of lysis buffer. The samples were then incubated for 10 min on ice, followed by a further centrifugation at 10000 x g for 5 min. While the pellet was discarded, the supernatant was placed into a new microcentrifuge tube and put on ice. Thereafter, a BCA™ assay was performed in order to obtain the supernatant’s protein concentration. This was followed by 200 μg of protein being diluted per sample, prior to being added to wells of a 96-well plate. Once this was completed, 20 μl of 5X assay buffer was added to every sample. Thereafter, 10 μl of peptide substrate was added followed by incubation at 37 °C for 2 h. Finally, the absorbance was read at 405 nm. Note: esiRNA-RLUC treated cells served as a negative control and PCA treated cells were used as a positive control.

### Statistical evaluation

In order for accurate data analysis, Student’s t-test had to be utilized, with a confidence interval of 95%. Furthermore, *p*-values greater than 0.05 were seen as non-significant. To measure the degree of association between LRP/LR levels and apoptotic induction as well as cellular viability, Pearson’s correlation coefficient was calculated. A positive coefficient shows a directly proportional relationship between the two variables (where values close to 1 indicates a highly positive correlation).

## Results

### siRNA technology successfully results in knock-down of LRP expression and reductions in cellular viability in early and late stage colorectal cancer cells

**Fig. 1:**
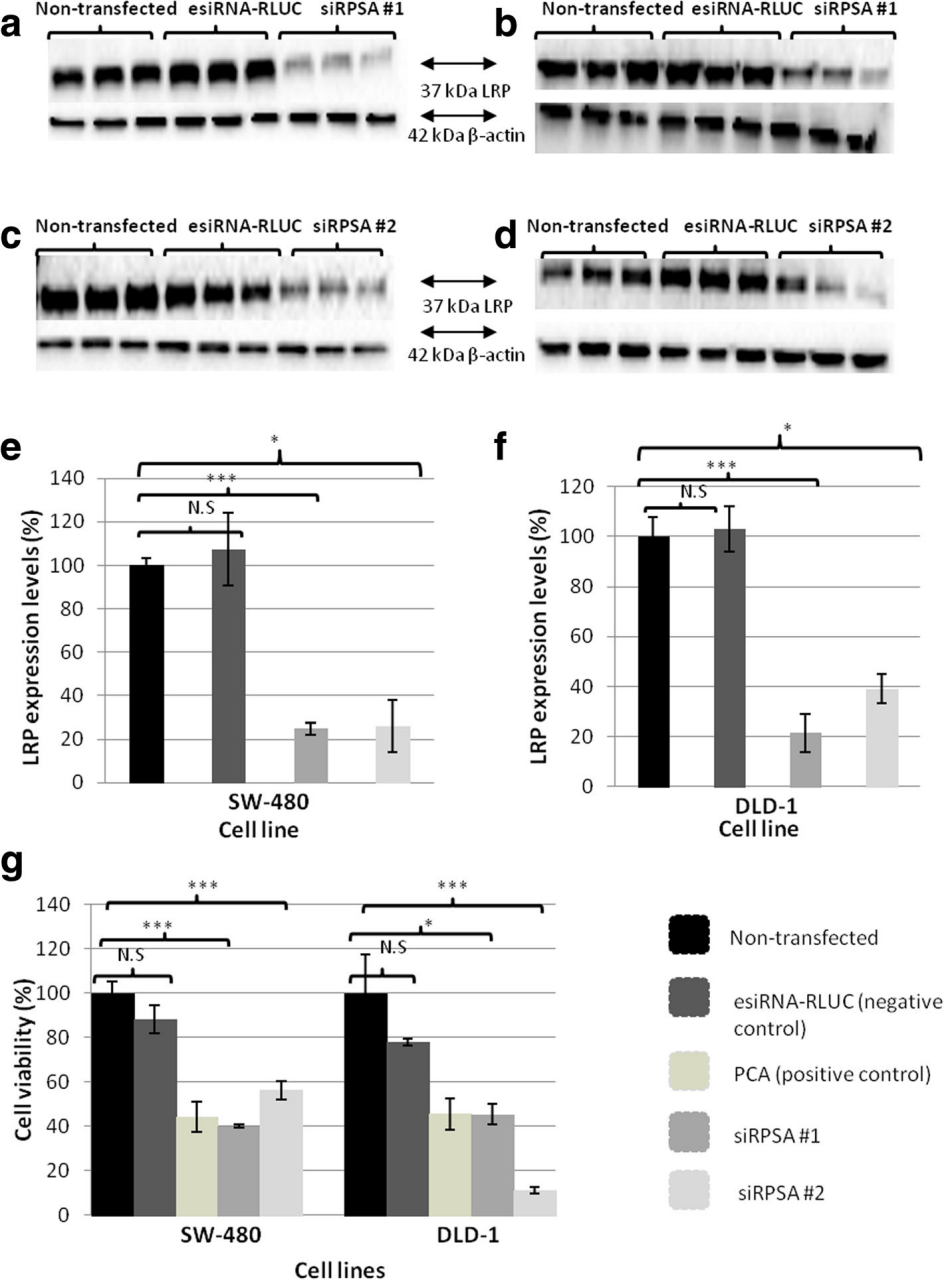
The effect of siRNA-mediated knock-down on LRP expression and the viability of early (SW-480) and late (DLD-1) stage colorectal cancer cells. **a**), **c**) and **e**) Upon transfection of SW-480 with siRPSA #1 and siRPSA #2, significant 75 and 72% decreases in LRP expression levels was revealed, respectively, in contrast to cells that were not transfected. Densitometric analysis of LRP levels was performed where levels of the non-transfected cells were set to 100%. ***p = 0.0001, *p = 0.02 N.S.:*p* > 0.05. **b**), **d**) and **f**) siRPSA #1 and siRPSA #2 transfection in DLD-1 cells caused significant 79 and 61% decreases in LRP expression levels, respectively, when comparing them to cells that were not transfected. Densitometry displayed no significant difference in LRP expression between non-transfected and negative control esiRNA-RLUC transfected cells for both cell lines. ***p = 0.0005, *p = 0.02 N.S.:p > 0.05. **g**) MTT assays were performed to assess SW-480 and DLD-1 cell viability, upon treatment with siRPSA #1 and siRPSA #2. Non-transfected cell value were set to 100%. It was found that upon siRPSA #1 transfection, SW-480 and DLD-1 cells exhibited a significant decrease of 60 and 55% in cellular viability, respectively, in contrast to cells that were not transfected. It was also revealed that when the SW-480 and DLD-1 were transfected with siRPSA #2, there were significant reductions of 44 and 89% in cellular viability, respectively, when compared to non-transfected cells. Both cell lines showed no noteworthy differences in cell viability when treated with the negative control esiRNA-RLUC. PCA was used as the positive control. SW-480: siRPSA #1:***p = 0.0008, siRPSA #2:***p = 0.0004 DLD-1: siRPSA #1:*p = 0.01, siRPSA #2:***p = 0.0009, N.S. :*p* > 0.05, non-significant. All graphs represent an average of three biological and three technical repeats

To understand the effect LRP/LR expression has on early and late stage colorectal cancer cell viability, down-regulation of the receptor had to be performed. Once early (SW-480) and late (DLD-1) cells were transfected with siRPSA #1 (targeted towards the 37 kDa LRP mRNA), evaluation of Western blotting and densitometry was performed. Densitometry showed that LRP was significantly knocked down in both SW-480 and DLD-1 cells when transfected with siRPSA #1. The SW-480 and DLD-1 transfected cells exhibited a 75 and 78% decrease in LRP expression, respectively, when compared to cells that were not transfected, since these LRP levels were set to 100% (Fig. 1a and b). Additionally, to determine whether the reduction in LRP expression had resulted due to siRPSA #1-mediated LRP knock-down and not just an off-target effect, an alternative siRNA that targets another region of LRP was utilised. When comparing them to the non-transfected cells, both early (SW-480) and late (DLD-1) stage colorectal cancer cells displayed a knock-down of 72 and 61% in LRP expression, respectively (Fig. 1c and d). SW-480 and DLD-1 cells transfected with the esiRNA-RLUC showed no significant LRP knock-down when comparing them to non-transfected cells. Once LRP expression was successfully down-regulated using siRNA technology, its effect on the viability of both cell lines were observed. MTT assays were performed which showed that when SW-480 and DLD-1 cells were transfected with siRPSA #1, cell viability was significantly decreased in contrast to cells that were not transfected, indicating that siRNA-mediated knock-down of the receptor leads to reductions in cell viability in both early and late stage colorectal cancer cell lines. The MTT assays revealed that SW-480 and DLD-1 cells treated with siRPSA #1 displayed a 60 and 55% decrease, respectively, in comparison to the cells that were not transfected (Fig. 1g). Additional MTT assays were performed to evaluate whether siRPSA #2-mediated LRP knock-down also influenced the viability of SW-480 and DLD-1 cells. Post treatment of cells with siRPSA #2 was found to reduce the viability significantly by 44 and 89% in both SW-480 and DLD-1 cells, respectively, when comparing them to the cells which were not transfected (Fig. 1g). Additionally, treating SW-480 and DLD-1 cells with the negative control siRNA, esiRNA-RLUC, indicated no notable change in cell viability in comparison to cells that were not transfected (Fig. 1g).

### Knock-down of LRP expression via siRNA technology leads to changes in nuclear morphology signifying apoptosis

**Fig. 2:**
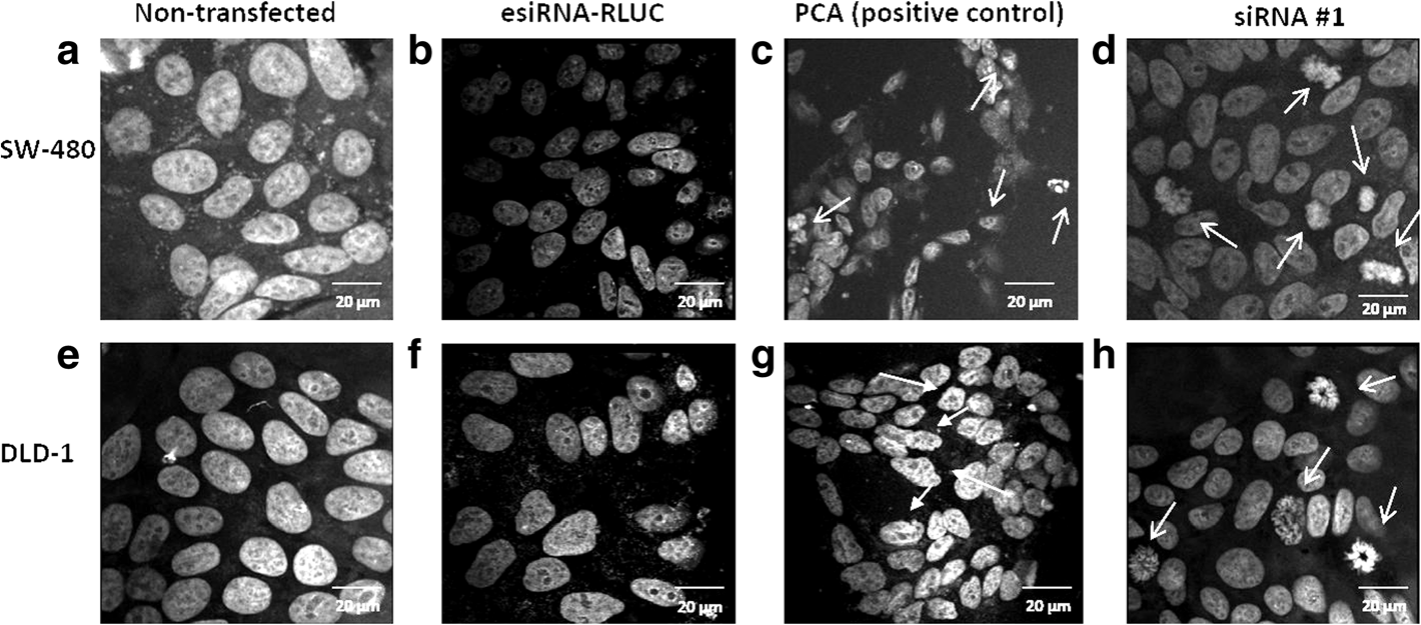
The effect of siRNA-mediated knock-down of LRP on nuclear morphology of early (SW-480) and late (DLD-1) stage colorectal cancer cells. Confocal microscopy with Airyscan analysis was completed to investigate changes in nuclear morphology upon siRNA treatment. **a** and **e**) Non-transfected SW-480 and DLD-1 cells displayed large nuclei with healthy membrane integrity. **b** and **f**) SW-480 and DLD-1 cells transfected with negative control esiRNA-RLUC showed parallel characteristics to the non-transfected cells with no changes in nuclear morphology. **c**) SW-480 cells treated with positive control PCA revealed nuclei that underwent apoptotic body formation. **d**) SW-480 cells transfected with siRPSA #1 exhibited nuclear shrinkage and condensed nuclei, proposing induction of apoptosis. **g**) DLD-1 cells treated with PCA displayed weakened membrane integrity and condensed nuclei. **h**) DLD-1 cells transfected with siRPSA #1 showed formation of apoptotic bodies and weakened membrane integrity. Images were obtained at a 630X magnification and Airy scan analysis was applied to each image. Scale bars are indicative of 20 μm

To determine whether the decreases in cell viability after treatment with siRPSA #1 was caused by apoptotic induction, nuclear morphological changes were studied by confocal microscopy and Airyscan. siRPSA #1 treated early (SW-480) stage colorectal cancer cells exhibited nuclear morphological changes in the form of condensed nuclei and reduced nuclear size (Fig. 2d), when compared to the nuclei of cells that were not transfected (Fig. 2a). Late (DLD-1) stage colorectal cancer cells transfected with siRPSA #1 presented nuclear morphological changes such as cellular fragmentation into membrane-bound bodies, weakened membrane integrity and membrane blebbing and (Fig. 2h), in contrast to nuclei of cells that were not transfected (Fig. 2e). Membrane blebbing was confirmed for the DLD-1 cells by bright field microscopy (Additional file 1: Figure S1). Results obtained for siRPSA #1 treated cells were consistent with those of the positive control, PCA (Fig. 2c and g). In addition, upon treatment with esiRNA-RLUC, both cell lines did not reveal any morphological changes in nuclei, when comparing them to cells that were not transfected (Fig. 2b and f).

### siRNA-mediated knockdown of LRP expression induces apoptosis in early and late stage colorectal cancer cells

Due to confocal microscopy proposing that the knock-down of LRP expression in early (SW-480) and late (DLD-1) stage colorectal cancer cells leads to morphological changes of the nuclei (a key feature of cells undergoing apoptosis), Annexin-V/PI assays had to be performed to confirm this result quantitively.

SW-480 cells treated with siRPSA #1 revealed 36.6% of cells undergoing early apoptosis, while 44.1% of cells underwent late apoptosis (Fig. 3.1d), in contrast to cells that were not transfected (Fig. 3.1a). Transfected DLD-1 cells with siRPSA #1 resulted in 10.0% of cells undergoing early apoptosis while 74.3% of cells underwent late apoptosis (Fig. 3.1h) when compared to cells that were not transfected (Fig. 3.1e) Additionally, upon treatment with esiRNA-RLUC, SW-480 and DLD-1 cell lines did not undergo apoptosis (Fig. 3.1b and f). PCA positive control showed most cells underwent late apoptosis (Fig. 3.1c and g. Further statistical analysis confirmed a significant increase in apoptotic cells when treated with siRPSA #1, in contrast to non-transfected cells in both cell lines (Fig. 3.2).

**Fig. 3:**
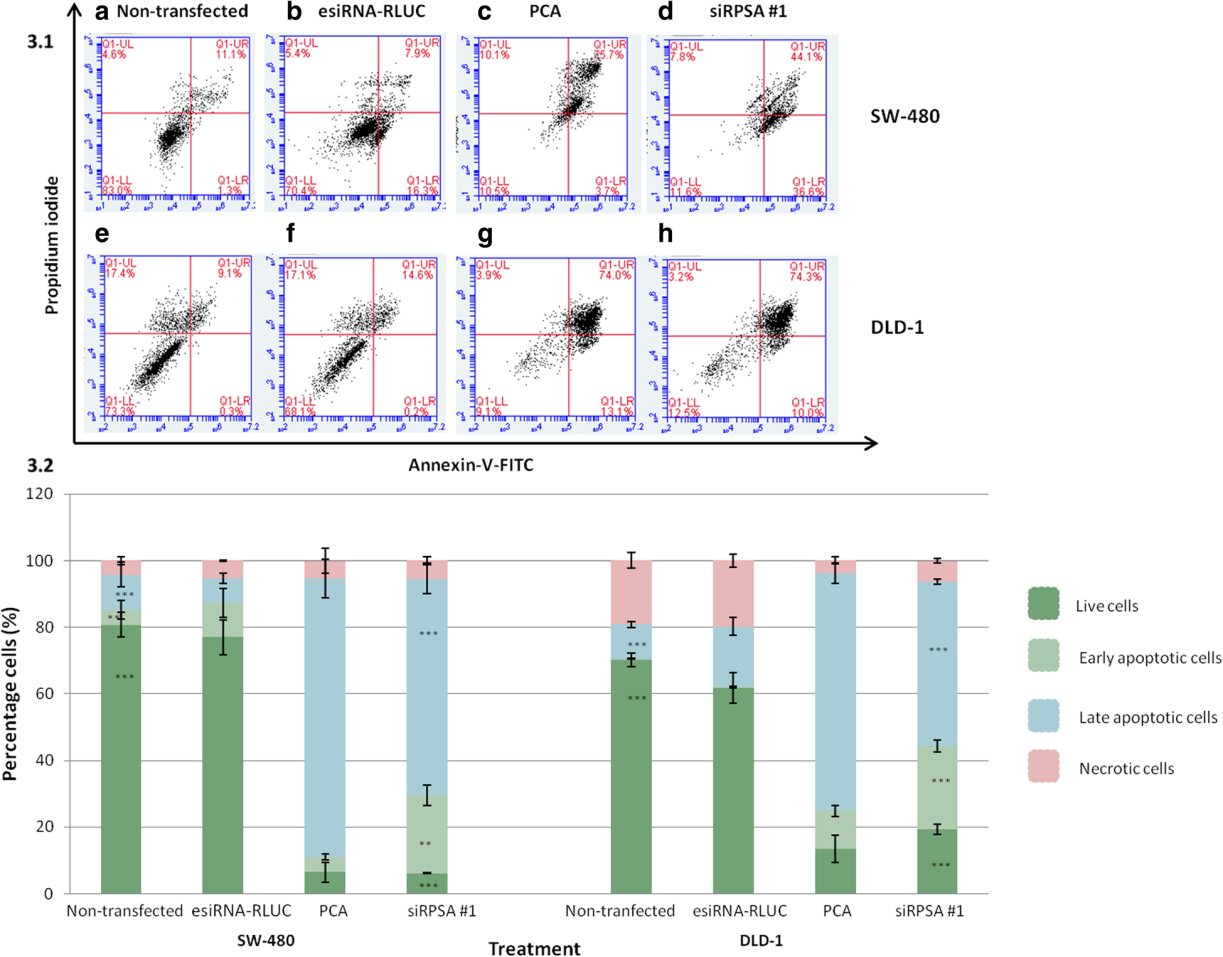
3.1 Apoptotic induction in early (SW-480) and late (DLD-1) stage colorectal cancer cells post siRNA transfection. It was revealed that most of the non-transfected **a**) SW-480 and **e**) DLD-1 cells fall in the lower quadrant (Q1-LL) which is known to represent normal live cells. **b**) and **f**) SW-480 and DLD-1 cells treated with negative control esiRNA-RLUC mostly appeared in the lower quadrant indicating live cells. **c**) and **g**) Positive control PCA, revealed that most cells were found in the upper right quadrant (Q1-UR), representing SW-480 and DLD-1 cells undergoing late apoptosis, respectively. **d**) and **h**) Cells transfected with siRPSA #1 resulted in 36.6% of SW-480 cells (**d**) and 10% of DLD-1 cells (**h**) undergoing early apoptosis, which is depicted in the lower right quadrant (Q1-LR); while 44.1% of SW-480 cells and 74.3% of DLD-1 cells underwent late apoptosis. This indicates that upon treatment with siRPSA #1, a total of 80.7% of SW-480 cells and 84.3% DLD-1 cells underwent apoptosis. 3.2 Bar graph illustrating apoptotic induction in early (SW-480) and late (DLD-1) colorectal cancer cells after siRNA transfection. This graph displays an average of three experiments completed in triplicate. Percentages for each quadrant were pooled together and compared to one another for both cell lines. It was found that SW-480 and DLD-1 cells had a significant increase in early and late apoptosis when treated with siRPSA #1, in contrast to cells that were not transfected, and both cell lines were seen to undergo more late apoptosis than early apoptosis. SW-480: ***p = 8.92029E-06 (live), ***p = 0.0001 (early apoptosis), **p = 0.002; DLD-1: ***p = 1.97127E-05 (live), ***p = 3.72195E-05 (early apoptosis), ***p = 1.08522E-06 (late apoptosis)

### siRNA-mediated knock-down of LRP expression causes a notable increase in caspase-3 activity

**Fig. 4:**
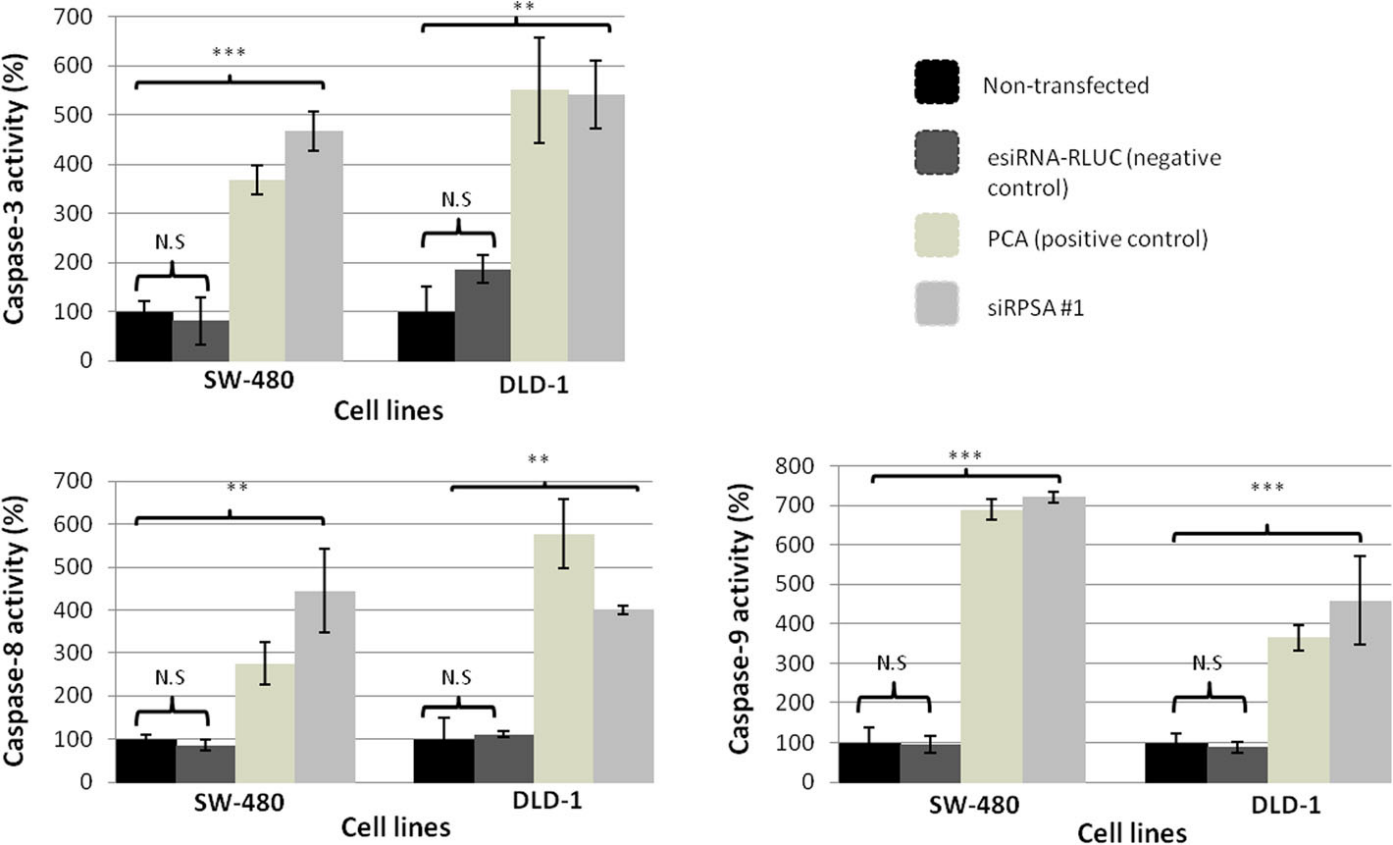
The effect of siRNA-mediated LRP knock-down on caspase-3, − 8 and − 9 activity in early (SW-480) and late (DLD-1) stage colorectal cancer cells. **a**) Upon treatment of SW-480 and DLD-1 cells with siRPSA #1, a significant 4-fold and 5-fold increase in caspase-3 activity was revealed, respectively, in comparison to cells that were not transfected (set to 100%). Both cell lines showed no significant difference in caspase-3 activity between cells transfected with negative control esiRNA-RLUC and non-transfected cells. PCA was used as a positive control. SW-480:***p = 0.0007 and DLD-1: **p = 0.0059. N.S: *p* > 0.05, non-significant. **b**) siRPSA #1 transfected SW-480 and DLD-1 cells both displayed a 4-fold significant increase in caspase-8 activity, compared to cells that were not transfected (set to 100%). Both cell lines showed no significant difference in caspase-8 activity between cells transfected with the esiRNA-RLUC and non-transfected cells. SW-480: **p = 0.0083 and DLD-1: **p = 0.002. N.S: *p* > 0.05, non-significant. **C)** SW-480 and DLD-1 cells transfected with siRPSA #1 showed a significant 7-fold increase and 4-fold increase, respectively, in contrast to cells that were not transfected. Both cell lines showed no significant difference in caspase-9 activity between cells transfected with the esiRNA-RLUC and non-transfected cells. SW-480:***p = 0.0001 and DLD-1: ***p = 0.0008. N.S.: *p* > 0.05, non-significant. This data represents three biological replicates which were completed in triplicate

For additional validation of apoptosis occurring in early (SW-480) and late (DLD-1) stage colorectal cancer cells once LRP has been down-regulated, caspase-3 activity assays were completed. Post transfection with siRPSA #1, SW-480 cells were found to have a significant 4-fold increase in caspase-3 activity, in comparison to cells that were not transfected (Fig. 4a). DLD-1 cells treated with siRPSA #1 displayed a 5-fold increase in caspase-3 activity in contrast to non-tranfected cells (Fig. 4a). Furthermore, no differences in caspase-3 activity was seen when both cell lines were treated with esiRNA-RLUC (Fig. 4a). PCA positive control displayed a 3-fold and 5-fold increase in caspase-3 activity was observed in SW-480 and DLD-1 cells, respectively (Fig. 4a).

### siRNA-mediated knock-down of LRP results in significant increases in caspase-8 and caspase-9 activity in early and late stage colorectal cancer cells

Caspase-3 activation occurs through both apoptosis pathways (intrinsic and extrinsic) hence, further insight of how the receptor aids in tumourigenic cell survival was required; therefore caspase-8 and -9 activity assays were performed. These assays determine whether treatment with siRPSA #1 leads to the activation of the extrinsic pathway (facilitated through caspase-8) or the intrinsic pathway (facilitated through caspase-9) in each of the cell lines. SW-480 and DLD-1 cells transfected with siRPSA #1 were found to undergo a 4-fold increase in caspase-8 activity, in comparison to cells that were not transfected (Fig. 4b). Moreover, SW-480 cells and DLD-1 cells indicated a significant 7-fold and 4-fold increase in caspase-9 activity, respectively, in comparison to cells that were not transfected (Fig. 4c). Moreover, both cell lines transfected with esiRNA-RLUC displayed no differences in caspase-8 and -9 activity in comparison to cells that were not transfected (Fig. 4b).

## Discussion

LRP/LR has gained a large amount of interest due to the many roles it plays in the cell. Particularly, the receptor’s over-expression in several cancer cell types as well as its contribution in tumourigenesis has become a target area for research. LRP/LR has been seen to assist with several tumourigenic processes including tumour adhesion and invasion (metastasis), angiogenic enhancement as well as apoptotic evasion [3]. Additionally, since LRP/LR is not limited to the cell surface but also localized in the perinuclear region, cytosol and nucleus, it is able to perform many intracellular and extracellular physiological roles including maintaining cell viability, cell adhesion, cell growth and migration, cell cycle regulation, ribosomal anchorage to microtubules, pre-rRNA processing and protein synthesis. Thus, cancerous cells are found to over-express LRP/LR, thereby exploiting these functions, resulting in the development of the abovementioned tumourigenic processes. Moreover, a recent study performed by Vania et al. showed that there were significantly higher levels of the receptor in late (DLD-1) stage colorectal cancer cells, compared to the early (SW-480) stage – indicating that LRP expression also increases in the course of malignant transformation [4].

To gain insight into how LRP/LR maintains cell viability, siRNA technology was used to knock-down LRP expression (Fig. 1) and evaluate this effect on cell viability of SW-480 and DLD-1 cells. Due to siRPSA #1 only targeting the mRNA of the 37 kDa laminin receptor precursor (LRP) form, it was employed to down-regulate LRP in this study (see Additional file 1: Table S1). Cells treated with siRPSA #1 resulted in significant decreases in LRP down-regulation. Furthermore, a high correlation of 0.91 for SW-480 and 0.96 for DLD-1 was observed between total levels of LRP before and after siRPSA #1 transfection (Table 1). This high and positive correlation suggests that the level of LRP expression is indeed influenced by siRNA treatment i.e. lower levels of LRP expression prior to treatment with siRNA leads to more LRP knockdown post treatment with siRNA.

**Table 1:** Assessment of correlation between levels of siRNA-mediated LRP knockdown and viability, apoptotic levels and caspase-3 activity of early (SW-480) and late (DLD-1) stage colorectal cancer cells, using Pearson’s correlation co-efficients (R)

Cell line	SW-480	DLD-1
Correlation between total LRP levels before and after siRPSA #1 transfection (R-value)	0.91	0.96
Correlation between siRPSA #1-mediated LRP knockdown and reduction in cell viability (R-value)	0.99	0.98
Correlation between siRPSA #2-mediated LRP knockdown and reduction in cell viability (R-value)	0.99	0.93
Correlation between total levels of apoptosis and total LRP levels after siRPSA #1 transfection (R-value)	0.99	0.98
Correlation between increases in caspase-3 activity and total LRP levels after siRPSA #1 (R-value)	0.94	0.93

To validate that the observed knock-down was not due to off target effects, SW-480 and DLD-1 cells were both treated with an alternative siRNA, siRPSA #2. This siRNA targets a specific region of 37 kDa LRP mRNA i.e. nucleotides 521–929 (Table 1). Upon treatment with siRPSA #2, both cell lines showed significant decreases in LRP knock-down in contrast to cells that were not transfected (Fig. 1). These results validated that LRP was being down-regulated and was not just an off-target effect. In addition, the correlation between total LRP levels before and after siRPSA #2 treatment was found to be high (sees Additional file 1: Table S2).

To further investigate the receptor’s role in maintaining cell viability, an MTT assay was employed to investigate the effect of treatment with siRPSA #1 and siRPSA #2 on cell viability. A significant decrease in viability was observed for both SW-480 and DLD-1 cells after LRP down-regulation (Fig. 1). These reductions in cellular viability correlate with the decreased levels of LRP observed after siRNA-mediated down-regulation, signifying the receptor’s vital role in the survival of SW-480 and DLD-1 cells (Table 1). It has been discovered that LRP/LR localised in the nucleus allows for chromosome stability maintenance via interactions with the Midkine heparin-binding growth factor; well-known for enhancing cell proliferation, migration and survival [36]. In addition, several cancer types are showed to have an up-regulated expression of Midkine, which results in the promotion of cell survival factors as well as obstruction of apoptosis through caspase-3 inhibition [37]. However, by targeting LRP expression through siRNA technology, LRP/LR-Midkine interactions may decrease, and as a result decrease cell viability.

To establish whether the observed decrease in SW-480 and DLD-1 cell viability post LRP knockdown was due to cell death caused by apoptosis, confocal microscopy was used to evaluate nuclear morphology. Both cell lines revealed several changes in nuclear morphology which were all characteristic of apoptosis including: nuclear condensation, reduced nuclear size and the formation of membrane-bound bodies – when treated with siRPSA #1 (Fig. 2). It is known that nuclear structures are maintained via the binding of histones to perinuclear and nuclear LRP/LR, thus when the receptor is down-regulated, loss of membrane integrity and distorted nuclear morphology is evident [38].

Although confocal microscopy provided a visual indication that apoptosis was occurring, additional quantification and affirmation of apoptotic induction was needed. This was made possible by means of Annexin V-FITC/PI assays. Non-transfected and negative control esiRNA-RLUC-transfected SW-480 and DLD-1 cells showed negative staining for Annexin-V – indicating live cells. On the other hand, siRPSA #1-treated and PCA-treated SW-480 and DLD-1 cells exhibited positive staining for Annexin-V or Annexin-V and PI, indicating early and late stage apoptosis, respectively. This shift of Annexin-V staining from negative to positive shows that siRPSA #1-mediated down-regulation in SW-480 and DLD-1 cells activates membrane asymmetry loss and a membrane-flip reaction involving the externalization of phosphatidylserine (PS) on the outer leaflet of the plasma membrane – allowing these cells to be taken up by phagocytes [39]. Thus, these findings together with the nuclear morphological changes observed, confirm that siRNA-mediated down-regulation of LRP/LR leads to the induction of apoptosis in SW-480 and DLD-1 cells. In addition, the correlation (Table 1) between total levels of LRP after siRPSA #1 treatment and total levels of apoptosis for both cell lines was found to be high (Figs. 1 and 3.1), further reiterating LRP expression affects cell viability and apoptosis.

Sustantad et al. found that when LRP/LR is down-regulated via siRNA technology, not only did the cell viability of cervical cancer (HeLa) cells and lung (A549) cancer cells decrease but caspase-3 activity was increased in both cell lines [27]. Caspase-3, an effector caspase, is responsible in executing the hallmarks of apoptosis which includes the afore-mentioned nuclear morphological changes by cleaving substrates [40]. Therefore, upon apoptotic induction, caspase-3 activity is found to be significantly increased. Hence, to further establish that apoptosis was indeed occurring and whether caspases were activated after treatment with siRPSA #1, caspase-3 assays were performed. Down-regulation of LRP caused a distinct increase in caspase-3 activity in SW-480 and DLD-1 cells when compared to the cells that were not transfected. Furthermore, the correlation between the total levels of LRP after siRPSA #1-mediated knockdown (Fig. 1) and increases in caspase-3 activity (Fig. 4) was high in both cell lines (Table 1). These results noticeably point to the induction of apoptosis in SW-480 and DLD-1 cells due to the silencing of LRP though siRNA technology.

It has previously been shown that interactions between LRP/LR and focal adhesion kinase (FAK) are made possible via the binding of the receptor to laminin-1. Furthermore, LRP/LR-FAK interactions were seen to be involved in activating cell signalling cascades such as MEK/ERK 1/2 and PI3-kinase/AKT as well as up-regulating the anti-apoptotic protein, Bcl-2 [41]. This ultimately leads to the inhibition of apoptosis of cancerous cells. Hence, we suggest that silencing LRP/LR through the use of siRNA technology as performed in the current study, impedes the LRP/LR-FAK interaction, and in this way apoptosis is induced. Furthermore, LRP/LR has been shown to have a direct relationship with the MAPK signalling pathway – where decreased levels of LRP causes a response in the pathway – resulting in cell stress and ultimately, cell death [10]. Another reason which could have led to apoptotic induction through siRNA-mediated knock-down of LRP is the receptor’s role in ribosomal processing. Research has shown that LRP/LR is involved in processing 21S pre-rRNA into mature 18S rRNA, also known as biogenesis of ribosomes [16]. LRP/LR has also been shown to associate with pre-40S ribosomal subunits, providing nucleolar exits for the subunits, thereby facilitating protein synthesis [42]. We propose that the LRP down-regulation performed in this study may have hampered formation of the ribosome as well as the resultant translation of proteins required for correct cellular functioning, ultimately leading to apoptosis. Furthermore, LRP has also been seen to play a key role in the cell cycle thus, down-regulating the receptor could have resulted in the induction of G1-phase arrest in the colorectal cancer cells, aiding in apoptosis [10].

Since both the MEK/ERK 1/2 and PI3-kinase/AKT cell signalling cascades are known to inhibit both apoptotic pathways, caspase-8 and caspase-9 assays were performed to determine if these caspases are activated upon siRPSA #1-mediated LRP/LR knock-down. Both early and late stage colorectal cancer cell lines were found to have higher caspase-8 activity post transfection with siRPSA #1, in contrast to cells that were not transfected. Caspase-8 plays a vital role in the extrinsic apoptotic signalling pathway through death receptors, thus it is suggested that siRNA-mediated LRP knock-down induces the apoptotic process in SW-480 and DLD-1 cells extrinsically. Moreover, this study revealed that SW-480 cells and DLD-1 also have increased in caspase-9 activity after LRP down-regulation, in contrast to cells that were not transfected. Caspase-9 plays a critical role in the intrinsic apoptotic signalling pathway, proposing that siRNA-mediated LRP knock-down also initiates apoptosis in SW-480 and DLD-1 cells through the intrinsic pathway.

The intrinsic and extrinsic pathways interconnect with each other at several levels and can both be influenced by similar factors. In fact, one study showed that activated extrinsic caspase-8 stimulated the release of cytochrome *c* and apoptosome formation and ultimately activation of the intrinsic pathway [43, 44]. A potential reason as to why SW-480 and DLD-1 cells experience apoptosis through both apoptotic pathways may be that these colorectal cancer cells undergo a mechanism known as retaliatory caspase activation where the two apoptotic pathways are found to use a feedback amplification loop in order to activate one another [45]. Specifically, activated caspase-9 initiates and proteolytically cleaves caspase-3, also leading to caspase-8 activation [45, 46]. Moreover, due to SW-480 and DLD-1 cells undergoing both apoptotic pathways, it can be said that down-regulated LRP/LR possibly hampers both anti-apoptotic signalling pathways on account of the reduced interaction of phosphorylated FAK and LRP/LR.

## Conclusions

This study shows that down-regulating LRP via siRNA technology significantly decreases the viability of early (SW-480) and late (DLD-1) stage colorectal cancer cells through the induction of apoptosis. Moreover, SW-480 and DLD-1 cells underwent apoptosis through both apoptotic pathways. It is possible that cell signalling cascades are involved in inducing apoptosis, however, the exact mechanism is unclear. These findings demonstrates the critical function LRP/LR plays in maintaining the viability of both early and late stage colorectal cancer cells. In addition, these findings emphasize the therapeutic potential of siRNAs targeted against LRP, which could be used as a possible tool in treating early and late stage colorectal cancer.

## Additional file

Additional file 1: **Figure S1.** Late stage (DLD-1) colorectal cancer cells show membrane blebbing and reduced nuclei post transfection with siRPSA #1 using bright field microscopy. A) and B) Non-transfected and esiRNA-RLUC (negative control) transfected cells are found to be large with uncompromised membrane integrity. C) and B) siRPSA #1-transfected and PCA (positive control) treated cells are found to have a reduced size together with compromised membrane integrity i.e. membrane blebbing and condensed nuclei – all indicative of apoptosis occurring. Images were obtained at 200X magnification. Scale bars are indicative of 20 μm. **Table S1.** Sequence of Human-RPSA, esiRNA-RPSA and control siRNA-RLUC used for down-regulation of LRP/LR. **Table S2.** Pearson’s correlation co-efficients (R) between total LRP levels prior to and post transfection with esiRNA-RPSA (DOCX 425 kb)

## Abbreviations

ATCC: American type culture collection
BCA: Bicinchoninic acid
BSA: Bovine serum albumin
CO_2_: Carbon dioxide
DMEM: Dulbecco’s Modified Eagle’s medium
DMSO: Dimethyl sulfoxide
ERK: Extracellular signal-regulated kinases
FAK: Focal adhesion kinase
FCS: Fetal calf serum
FITC: Fluorescein isothiocyanate
HRP: Horseradish peroxidase
IgG: Immunoglobulin G
kDa: Kilodaltons
LRP/LR: Laminin receptor precursor/ laminin receptor
MTT: 3-(4,5-dimethylthiazol-2-yl)-2,5-diphenyltetrazolium bromide
PAGE: Polyacrylamide gel electrophoresis
PBS: Phosphate buffered saline
PCA: Protocatechuic acid
PI: Propidium iodide
PI3K: Phosphoinositide 3-kinase
PS: Phosphatidyl serine
PVDF: Polyvinylidene fluoride
RLUC: Renilla luciferase
RNA: Ribonucleic acid
Rpm: Revolutions per minute
RPSA: Ribosomal protein SA
SDS: Sodium dodecyl sulfate
siRNA: Small interfering RNA
TEMED: Tetramethylethylenediamine
