# Supplementary material for: Knock-down of LRP/LR promotes apoptosis in early and late stage colorectal carcinoma cells via caspase activation
Source: BMC Cancer. 2018 May 29;18:602. doi: 10.1186/s12885-018-4531-2 (PMC5975593; doi:10.1186/s12885-018-4531-2)
Supplement: Supplementary file 1 — Figure S1. Late stage (DLD-1) colorectal cancer cells show membrane blebbing and reduced nuclei post transfection with siRPSA #1 using bright field microscopy. A) and B) Non-transfected and esiRNA-RLUC (negative control) transfected cells are found to be large with uncompromised membrane integrity. C) and B) siRPSA #1-transfected and PCA (positive control) treated cells are found to have a reduced size together with compromised membrane integrity i.e. membrane blebbing and condensed nuclei – all indicative of apoptosis occurring. Images were obtained at 200X magnification. Scale bars are indicative of 20 μm. Table S1. Sequence of Human-RPSA, esiRNA-RPSA and control siRNA-RLUC used for down-regulation of LRP/LR. Table S2. Pearson’s correlation co-efficients (R) between total LRP levels prior to and post transfection with esiRNA-RPSA (DOCX 425 kb) [file 12885_2018_4531_MOESM1_ESM.docx]

**Additional file 1**

**
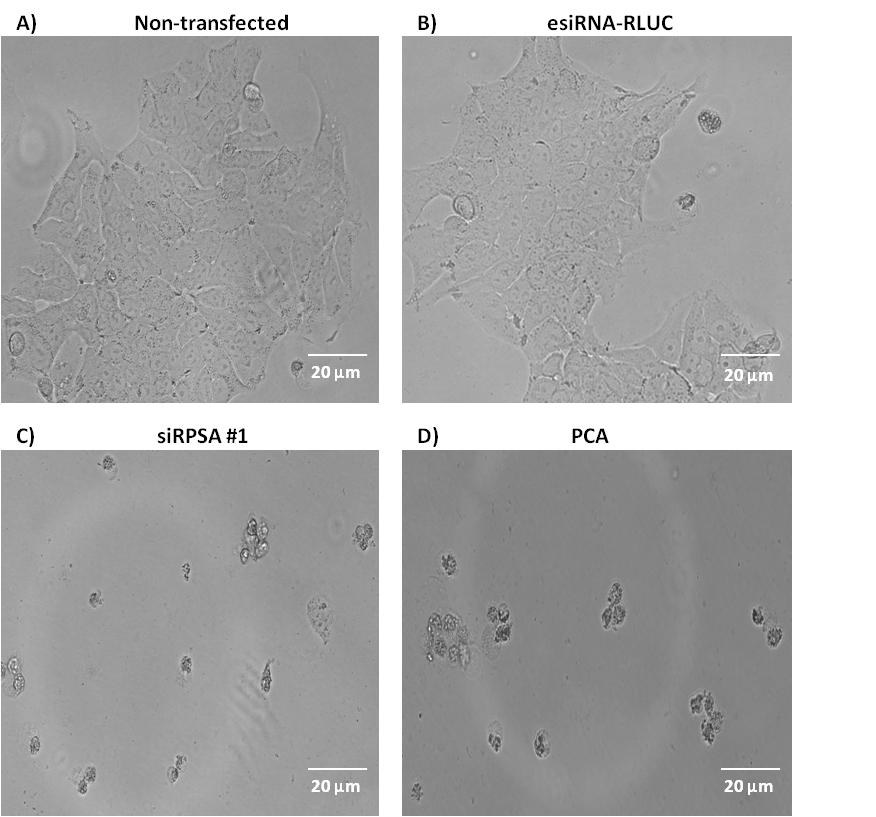
**

**Figure S1: Late stage (DLD-1) colorectal cancer cells show membrane blebbing and reduced nuclei post transfection with siRPSA #1 using bright field microscopy. A) and B)** Non-transfected and esiRNA-RLUC (negative control) transfected cells are found to be large with uncompromised membrane integrity. **C) and B)** siRPSA #1-transfected and PCA (positive control) treated cells are found to have a reduced size together with compromised membrane integrity i.e. membrane blebbing and condensed nuclei – all indicative of apoptosis occurring. Images were obtained at 200X magnification. Scale bars are indicative of 20 µm.

**Table S1: Sequence of Human-RPSA, esiRNA-RPSA and control siRNA-RLUC used for down-regulation of LRP/LR.**

| **siRNA** | **Sequence** | **Transfection reagent** |
| --- | --- | --- |
| ON-TARGETplus SMARTpool human-RPSA | 4 RPSA siRNA pooled together (targets 4 regions of LRP/LR):  Target sequence 1:  CGACAUGAGUUGUACUUCU  Target sequence 2:  GAUUGCAUAUCAAAGCAUA  Target sequence 3:  GGUCAUGCCUGAUCUGUAC  Target sequence 4:  UAUCAUAAAUCUCAAGAGG | DharmaFect1 |
| esiRNA-RPSA | CCTCTCACGGAGGCATCTTATGTTAACCTACCTACCATTGCGC  TGTGTAACACAGATTCTCCTCTGCGCTATGTGGACATTGCCAT  CCCATGCAACAACAAGGGAGCTCACTCAGTGGGTTTGATGTGG  TGGATGCTGGCTCGGGAAGTTCTGCGCATGCGTGGCACCATTT  CCCGTGAACACCCATGGGAGGTCATGCCTGATCTGTACTTCTA  CAGAGATCCTGAAGAGATTGAAAAAGAAGAGCAGGCTGCTGCT  GAGAAGGCAGTGACCAAGGAGGAATTTCAGGGTGAATGGACT  GCTCCCGCTCCTGAGTTCACTGCTACTCAGCCTGAGGTTGCAG  ACTGGTCTGAAGGTGTACAGGTGCCCTCTGTGCCTATTCAGCA  ATTCCCTACTGAAGACTGGAGCG | Mission transfection reagent |
| esiRNA-RLUC | GATAACTGGTCCGCAGTGGTGGGCCAGATGTAAACAAATGAAT  GTTCTTGATTCATTTATTAATTATTATGATTCAGAAAAACATGC  AGAAAATGCTGTTATTTTTTTACATGGTAACGCGGCCTCTTCT  TATTTATGGCGACATGTTGTGCCACATATTGAGCCAGTAGCGC  GGTGTATTATACCAGACCTTATTGGTATGGGCAAATCAGGCAA  ATCTGGTAATGGTTCTTATAGGTTACTTGATCATTACAAATAT  CTTACTGCATGGTTTGAACTTCTTAATTTACCAAAGAAGATCAT  TTTTGTCGGCCATGATTGGGGTGCTTGTTTGGCATTTCATTAT  AGCTATGAGCATCAAGATAAGATCAAAGCAATAGTTCACGCTG  AAAGTGTAGTAGATGTGATTGAATCATGGGATGAATGG | Mission transfection reagent |

**Table S2: Pearson’s correlation co-efficients (R) between total LRP levels prior to and post transfection with esiRNA-RPSA.**

| **Cell line** | **Correlation between total LRP levels prior to and post esiRNA-RPSA transfection (R-value)** |
| --- | --- |
| SW-480 | 0.98 |
| DLD-1 | 0.99 |

**Transfection procedure using Human-RPSA:**

Lyophilized Human-RPSA (5nmol/20nmol) was reconstituted in 100µl or 250μl of 1X RNAse free siRNA buffer, respectively, before use in order to make a 20 μM stock. The table below shows amounts of siRNA and corresponding components used for transfections of a 6-well plate.

| **Reagent** | **Volume (μl)** |
| --- | --- |
| Opti-MEM media – for addition of esiRNA | 190 |
| Human-RPSA | 10 |
| Opti-MEM media – for addition of transfection reagent | 190 |
| DharmaFect transfection reagent | 10 |

Transfection procedure:

Reconstituted Human-RPSA was added to the corresponding volume of Opti-MEM media in a micro centrifuge tube. In a second tube, transfection reagent was added to the corresponding volume of Opti-MEM media. Both tubes were incubated for 5 minutes at room temperature and mixed together. The resultant siRNA solution was further incubated for 20 minutes at room temperature.

**Transfection procedure using esiRNA-RPSA and esiRNA-RLUC:**

esiRNA-RPSA and esiRNA-RLUC are purchased reconstituted. The table below shows volumes of esiRNA and corresponding components used for transfections of a 6-well plate.

| **Reagent** | **Volume (μl)** |
| --- | --- |
| Opti-MEM media – for addition of esiRNA | 250 |
| esiRNA-RPSA or esiRNA-RLUC | 5 |
| Opti-MEM media – for addition of transfection reagent | 250 |
| Mission transfection reagent | 5 |

Transfection procedure:

esiRNA-RPSA or esiRNA-RLUC was added to the corresponding volume of Opti-MEM media in a micro centrifuge tube. In a second tube, transfection reagent was added to the corresponding volume of Opti-MEM media. Both tubes were incubated for 5 minutes at room temperature and the contents of both tubes were mixed together.

**Antibodies and siRNAs – Suppliers list:**

- IgG1-iS18 – Affimed Therapeutics
- Anti-human IgG-HRP, anti-human PE, anti-rabbit APC– Abcam
- Anti-β actin peroxidase, esiRNA-RPSA (200ng/ul), esiRNA-RLUC (200 ng/ul) – Sigma
- Human-RPSA siRNA(5 nmol/20nmol) and DharmaFect1 transfection reagent – GE Dharmacon

**Kits – suppliers list:**

- Annexin V-FITC/ PI kit – BD Biosciences
- Caspase 3,-8 and -9 kits and cell cycle kit – Merck Millipore

**Equipment list:**

- Flow cytometer – BD Accuri C6
- Confocal microscope – Zeiss LSM 710 3-channel (images were captured using the blue laser, 63X magnification, and using Zen 2011 software).
- Bright field microscope – Floid cell imaging station
- Laminar flow – Labotec
- ELISA reader – Tecan (using Magellan software)
- Centrifuge – Eppendorf 5417C
- Pipettes and micropipettes – Eppendorf research
- TC20 cell counter, gel casting and running apparatus – Biorad
